# Supplementary material for: Clinical and growth outcomes of severely malnourished children following hospital discharge in a South African setting
Source: PLoS One. 2022 Jan 21;17(1):e0262700. doi: 10.1371/journal.pone.0262700 (PMC8782382; doi:10.1371/journal.pone.0262700)
Supplement: Attachment — Submitted filename: Response to reviewers.docx [file pone.0262700.s001.docx]

PLOS One Rebuttal letter: Responses to reviewers

| Comment number | Reviewer comment | Authors response | Tracked changes |
| --- | --- | --- | --- |
| Reviewer #1 | | | |
| 1. | Omitting HFA <-3 as a criterion is appropriate as the causes and outcomes of stunting are largely not the same as those causing low WFL, MUAC and oedema. | Agree. HFA (stunting) was not the main interest of this study. | No changes to be made. |
| 2. | The core of the paper relates to nutritional outcomes including the welcome outcome of no post-discharge mortality. The link of nutritional outcome to clinical status at discharge is a useful finding, given that discharge criteria in the two institutions did not coincide with WHO recommendation. **I would recommend that these are recorded as planned secondary outcomes in the methods section.** Only mortality is mentioned there, yet the bulk of the article is about other nutritional outcomes. | Agree that further clarity is needed about the primary and secondary outcomes in the abstract and introduction of the paper. | **Abstract: (Line 11-13) – addition:** Secondary outcomes included relapse rates, type(s) and frequency of morbidities experienced and the anthropometric changes in children with SAM following hospital discharge  **Introduction: (Line 56-62) - replacement** “This study aimed to prospectively establish mortality and relapse rates, the frequency and type(s) of morbidities experienced, and anthropometric changes in children with SAM following hospital discharge. A secondary aim was to identify factors associated with poorer long-term growth, particularly those that might be amenable to change or that could assist in identifying children requiring more targeted support. It also explored the uptake and benefit of local SAM support services”  was **changed to**  “The study **primarily** aimed to establish mortality rates prospectively in children with SAM following hospital discharge. **Secondary outcomes** included relapse rates, the frequency and type(s) of morbidities experienced, and anthropometric changes in these children. It further aimed to identify factors associated with poorer long-term growth, particularly those that might be amenable to change or that could assist in identifying children requiring more targeted support. Lastly, it explored the uptake and benefit of local SAM support services. |
| 3. | The persistence of SAM and significant nutritional deficits is an important finding, though muddied by the HIV and CP groups who did not do well. While I buy the idea that they should be included, **reviewing the data without them** might give more insight into standard patients with SAM who are the bulk of the cases in secondary and primary care situations which is where bang for buck in improved f/u strategies is to be found at population level. | While we acknowledge that there may be benefits to restricting the study to nutritionally related SAM, the reality in southern Africa is that SAM often occurs secondary disease to HIV (up to half of the SAM cases previously) or disability such as CP.  We compared outcomes of children without HIV or CP, to those who had either of these conditions. We recognise that the failure to find statistically significant differences between the two groups relate to the small sample size. | **Additions to the manuscript** **(lines 233-235)**  No statistically significant differences in relapse rates, anthropometric outcome at three months and illness patterns were noted when comparing children who had SAM but not HIV or CP, to those who had either of these conditions. |
| 4. | While the paper gives extensive detail on discharge plans, nutritional inputs, follow up activities etc., including community follow up (rather dismal, it seems, and it does seem strange that with such small numbers involved one can discern that CHWs tended to worsen the situation. They may of course have only gone to those most at risk because the referring system made this more likely), because these are not standardised or grouped, their meaning in the context of the outcomes can't really be discerned for the large part - **thus these detailed data are superfluous to the main purpose of the article** (i.e. they don't provide guidance to others dealing with issues of SAM follow up - except to say that a more consistent strategy could be useful). | Although we understand the reviewer’s concern that some detail offered in the paper may seem superfluous, we believe that this additional detail strengthens the contribution of the paper.  The additional detail offers critical insights about some of the determinants of important outcomes such as relapse rates. This type of detail has been lacking in many previous studies on the topic preventing meaningful interpretation of reasons for differences in outcomes. | No changes were made. |
| 5. | Line 292 is an exception to this - **though some statistical evidence of the non-association of nutritional supplements with nutritional outcomes would is required**. This line is also misplaced - it should surely be under the Nutritional supplements heading (Line 299). | Agree and moved this statement. | “Receiving any nutritional supplements at one or three months was not associated with a reduced or higher risk of persistent malnutrition” was **moved to the “Nutritional Supplements” section (lines 318 - 319)** |
| 6. | You say in the Conclusion that 'The study has provided evidence that continuing nutritional support through supplementation may improve survival and reduce relapse rates in children with SAM following hospital discharge'. I suppose the key is word ''may' given line 291 which does not support statement this statistically (Type 2 error?). You are saying that the pervasiveness of supplementation is likely to be why there were not deaths and **many children improved nutritionally, but the study does not prove this unfortunately..** | We accept that the conclusion as presented may be construed as supporting the argument that nutritional supplements were **responsible** for the zero mortality and low relapse rate. At best they could have contributed to these outcomes. | The study has provided evidence that continuing nutritional support through supplementation may improve survival and reduce relapse rates in children with SAM following hospital discharge.” Removed from the conclusion.  **Addition in Conclusion (line 498):** “Further investigation of ideal nutritional supplementation programmes post discharge must be investigated.” |
| 7. | Comments are made re stunting but HFA does not appear among the measures during f/u in the Methods. | This detail has been added. | **Additions in (line 156-157) under Data analysis** “WAZ and HAZ” have been added.  Also, **additions in follow up section**:  Secondary growth measurements of underweight and stunting were measured using weight-for-age (WAZ) and height/length-for-age (HAZ). (**Line 143-145)** |
| 8. | The lack of association with variables listed towards the end of the Results section are likely to relate to small numbers as partial follow up data attrition started to bite (as you acknowledge in the Discussion). | We agree with the comment. As noted, we acknowledged this in the Discussion. | No changes made |
| 9. | Because this is a small sample (even though complete) in one region, I am recommending a revision to a short report, removing much of the dietary and other f/u detail which cannot be directly linked in a clear fashion to the nutritional outcomes (the main value of your work). (If you had been evaluating adherence to standard pre-discharge and f/u care of children with SAM, these details would have had more value.) | As discussed previously under #4, we believe that the additional data has merit.  We would be open to an editorial decision recommending a reduction in the word count.  We note that PLOS One does not offer short reports |  |
| Reviewer #2 | | | |
| 10. | This paper describes post-discharge outcomes of children receiving inpatient treatment for severe acute malnutrition. This is an area of limited research, and hence this paper makes a useful contribution to the literature, particularly due to the 6 months of follow-up provided and unique setting of an UMIC of South Africa. | Thank you for the comment. | No changes are necessary. |
| 11. | I recommend the paper be accepted after minor revision. This mainly relates to line 478 that the study 'has provided evidence that continuing nutritional support through supplementation may improve survival and reduce relapse rates in children with SAM following hospital discharge'. However it is not clear enough that the data supports this statement. There are many other results, such as the finding that better anthropometry at discharge is related to improved outcomes at follow-up, that could replace this. | This comment is similar to one made by Reviewer 1 and has been addressed in Comment #5. |  |
